# A New Approach to ORB Acceleration Using a Modern Low-Power Microcontroller

**DOI:** 10.3390/s25123796

**Published:** 2025-06-18

**Authors:** Jorge Aráez, Santiago Real, Alvaro Araujo

**Affiliations:** B105 Electronic Systems Lab, Escuela Técnica Superior de Ingenieros de Telecomunicación, Universidad Politécnica de Madrid, Avenida Complutense 30, 28040 Madrid, Spain; sreal@b105.upm.es (S.R.); araujo@b105.upm.es (A.A.)

**Keywords:** ORB, ARM microcontroller, SLAM, low-power microcontroller, visual odometry

## Abstract

A key component in visual Simultaneous Location And Mapping (SLAM) systems is feature extraction and description. One common algorithm that accomplishes this purpose is Oriented FAST and Rotated BRIEF (ORB), which is used in state-of-the-art SLAM systems like ORB-SLAM. While it is faster than other feature detectors like SIFT (340 times faster) or SURF (15 times faster), it is one of the most computationally expensive algorithms in these types of systems. This problem has commonly been solved by delegating this task to hardware-accelerated solutions like FPGAs or ASICs. While this solution is useful, it incurs a greater economical cost. This work proposes a solution for feature extraction and description based on a modern low-power mainstream microcontroller. The execution time of ORB, along with power consumption, are analyzed in relation to the number of feature points and internal variables. The results show a maximum of 0.6 s for ORB execution in 1241 × 376 resolution images, which is significantly slower than other hardware-accelerated solutions but remains viable for certain applications. Additionally, the power consumption ranges between 30 and 40 milliwatts, which is lower than FPGA solutions. This work also allows for future optimizations that will improve the results of this paper.

## 1. Introduction

The ability to locate and track the motion of objects, vehicles, or even individuals is a fundamental aspect of fields as diverse as logistics (e.g., Wi-Fi fingerprint location for Industry 4.0 [1]), emergency services (e.g., embedded in mobile network tracking [2]) autonomous vehicle development (e.g., through 5G-powered positioning [3]), or guidance systems for blind and visually impaired persons [4].

All these systems rely on specific technical approaches, ranging from beacon-based multilateration and triangulation [5] to fingerprinting, which involves mapping on-board sensor measurements to positions within a known environment (e.g., [6]). The choice of a specific solution requires leveraging system specifications (e.g., accuracy, coverage, or power consumption), application requirements (e.g., indoor environments), as well as deployment and maintenance costs.

Within this field, one of the most challenging scenarios is the development of standalone tracking devices capable of operating in unknown environments. In the absence of beacons or pre-registered data to serve as spatial references, these devices must rely solely on onboard sensors to track their movement. To overcome these limitations, prior solutions such as odometry and dead reckoning and later Simultaneous Localization and Mapping (SLAM) have been developed.

Specifically, as its name implies, SLAM systems gather and organize spatial data from the surroundings, maintaining the device’s position within an ever-growing internal map of the environment. In contrast, odometry and dead reckoning rely on differential data of the device’s position and orientation, usually gathered with cameras (visual odometry, e.g., [7]) in combination with inertial measurements (e.g., [8]). While these approaches are susceptible to accumulated drift error due to their reliance on integration, SLAM compensates for drift by leveraging loop closure when the device revisits previously registered areas.

Overall, SLAM implementations typically require real-time processing of sensor data, such as video streams and time-of-flight (ToF) measurements. As a result, their deployment on resource-constrained platforms, such as those used in robotics [9], has traditionally been challenging [10]. However, recent advancements in microcontroller processor architectures, memory capacities, and cost-efficiency have enabled even video-based, battery-powered applications.

Given how recent these advancements are, research is going in the direction of implementing algorithms that were traditionally meant to run on relatively powerful devices such as a PC in low-power microcontrollers, for example, machine learning models [11]. However, this trend has not applied to SLAM services, where no work on this matter has been found.

In this context, the objective of this research is to explore the feasibility of implementing SLAM solutions on current resource-constrained hardware platforms and an analysis of the time and resources consumed. Specifically, it describes the implementation of an ORB feature extraction and description module as the foundational step of an ORB-SLAM system and analyzes the tradeoff between system specifications and resource utilization.

Regarding the structure of the article, the next section will discuss the current implementation of ORB and the underlying decision-making process. In this context, the number of extracted feature points will be identified as a key factor in balancing system performance and resource utilization. Subsequently, power consumption, frames per second (FPS), and memory usage will be analyzed in relation to the number of extracted feature points. For benchmarking purposes, the KITTI database will be employed.

## 2. Materials and Methods

### 2.1. SLAM Approach for Resource-Constrained Platforms

When implementing SLAM on a low-resource platform, two aspects must be taken into account:

- Data source: SLAM can be visual or based on Light Detection And Ranging (LiDAR) measurements. Visual SLAM relies on one or more cameras as the source of information, while LiDAR uses specific hardware to record the distance from the sensor to an obstacle in all directions.
- Map detail: SLAM algorithms can be dense or sparse. Dense algorithms are notably more precise than sparse ones and create very detailed reconstructions of the environment. In contrast, sparse algorithms are lighter in terms of their memory and computation power requirements.

Having the objective of the work in mind, LiDAR is not suitable for the proposed used case, and sparse maps are computationally less expensive, which makes them suitable for a microcontroller. Considering these distinctions, the most fitting approach is a visual sparse algorithm. ORB-SLAM [12] has been used for this implementation, as it is a sparse algorithm that correctly fits the proposed constraints.

This algorithm comprises three main software components: visual odometry, local mapping, and loop closing. In a similar manner, each of these is subdivided into smaller submodules. Existing works [13,14] have addressed the task of bringing ORB-SLAM to the embedded world through hardware acceleration of the first step: ORB [15] computation from the image.

Visual odometry using ORB consists of analyzing frames for feature points, relating feature points of two different frames, and computing the displacement between images from these relationships. ORB consists of two steps: computing the position of the feature points and describing each feature point. To complete the visual odometry process, feature points in two different frames are corresponded between images, and the displacement between frames is obtained from epipolar geometry or bundle adjustment.

ORB comprises two main modules:

- Features from Accelerated Segment Test (FAST): This first module processes a grayscale image to detect feature points. The algorithm evaluates for each pixel of the image the intensity of the pixels in a circle of radius 3 around the evaluated point and compares it to the intensity of the middle point. If there are N consecutive pixels with brightness differences exceeding a specified threshold, the middle point is considered a feature point. The brightness difference is compared to a user-defined threshold. Additionally, non-maximal suppression can be used to discard feature points that are too close to one another. Depending on the number of consecutive points that are considered (N), the name of the algorithm will change (FAST9, FAST10…). For this work, the FAST implementation that was used is [16]. Specifically, The FAST-12 version with non-maximal suppression was used. The reason for using FAST-12 is that [16] includes an optimization that makes it possible to not consider all of the pixels around a potential feature point in order to discard it (refer to [17] Section 6.1.1 for the explanation), which results in a slight acceleration of the whole process.
- FAST orientation and Binary Robust Independent Elementary Features (BRIEF) descriptor: For each feature point, this module calculates an orientation and then a rotation-aware BRIEF description. The orientation is calculated using the intensity centroid method. Rotation-aware BRIEF accounts for orientation to compute the descriptor. A 256-bit descriptor length was implemented following the code proposed by [17].

In the remainder of this paper, the first and second module will be referred to as FAST and o+rBRIEF, respectively.

This paper aims to characterize the execution of the ORB algorithm, excluding hardware-related aspects like the acquisition of the images. For this reason, the tests that were performed assume that images are pre-acquired and focus solely on characterizing the different algorithms that conform ORB.

To evaluate the feasibility of ORB in resource-constrained environments, the algorithm has been implemented twice: one version for execution on the microcontroller and another for execution on a PC. Both implementations share the same code base and differ in how the input and outputs data are handled. Both implementations input the same images, which are available as part of the KITTI dataset [18]. This was meant to provide a specific scenario that could be compared to other previous implementations of ORB, instead of using our own images. This specific dataset has been chosen so as to use the same images as in [14].

### 2.2. Materials

For the embedded version, the microcontroller used was the STM32U575ZIT6-Q from STMicroelectronics (Geneva, Switzerland). More specifically, the NUCLEO-U575ZI-Q development board (STMicroelectronics, Geneva, Switzerland) was employed. The architecture of the microcontroller is Arm® Cortex®-M33, 32-bit. It has 2 MB of embedded Flash memory and 768 kB of embedded Static Random-Access Memory (SRAM). The operating frequency was configured to 160 MHz, the maximum allowed by the vendor. This specific microcontroller was chosen because it is a recent, ultra-low-power and low-cost microcontroller that is widely used in industry applications.

The PC implementation of ORB was executed on an HPE ProLiant server running a virtual machine with the software VMWare (version ESXi 6.7.0). The server uses an Intel® Xeon® E5-2470 v2 processor, with 8 virtual cores allocated to the virtual machine, running at 2.4 GHz. The virtual machine has 32 GB of DDR3 RAM memory assigned, along with 100 GB of HDD storage. The virtual machine OS is Ubuntu 22.04.3 LTS.

Power consumption was measured using a Keysight B2901A Precision Source and Measurement Unit (Keysight Technologies Inc., Santa Rosa CA, USA). To control the device, the Quick-IV Measurement Software (version 4.2.2045.2760) provided by the manufacturer was used.

ORB implementations for both the microcontroller and the PC have been written in plain C. The device implementations were as follows:

- For the microcontroller implementation, the software STM32CubeIDE (version 1.11.2) was used to develop all the necessary code for ORB to run on the microcontroller, which also handled compilation and debugging of the code. For compilation and debugging, the included GNU Toolchain for STM32 was used. The compiler was configured with -Ofast optimization and enabled the available Floating-Point Unit (FPU).
- For the PC implementation, the code was configured using CMake and built using gcc 11.4.0 as the compiler. The compiler was configured to use -O3 optimization.

The input images used for testing have been obtained from batch 0 of the KITTI dataset [18]. These images are grayscale, with 1 byte per pixel, and have a resolution of 1241 × 376 pixels.

### 2.3. Methods

#### 2.3.1. Measurements in PC

The execution time of ORB for each image was measured on a PC to be used as a reference point for comparison with the microcontroller.

The images that were employed had the same resolution for the whole batch. For this reason, resolution remained unmodified for the different tests that were performed. The parameters that were modified between images were the image itself and the FAST threshold value. The execution time is presented as a function of the latter. Less restrictive threshold values are expected to increase execution time, due to the optimization that was mentioned at the start of Section 2: The FAST-12 implementation that this paper uses can discard a potential feature point faster than it can accept it.

The procedure for measuring execution time on the PC was performed as follows:

1. Each grayscale image was converted to a byte array using a Python (version 3.12.2) script.
2. The byte array was included in the C project and processed by the two main functions: FAST and o+rBRIEF.
3. Timestamps at the start and at the end of each function were logged at the end of the processing of each image. These were recorded in millisecond scale.

**Figure 1:**
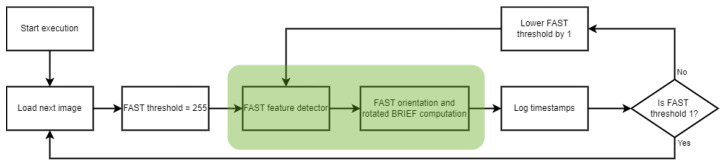
Flow diagram of the procedure for obtaining execution time measurements using the PC. Highlighted in green are the steps of the code that this work aims to characterize.

All of the combinations of image and FAST threshold were processed. A diagram of the process is shown in Figure 1.

Logs were processed, and execution times for each image were extracted. These results were compared to measurements obtained for the same images in the microcontroller.

#### 2.3.2. Execution Time Measurements in Microcontroller and Memory Usage Characterization

Execution time of ORB on the microcontroller was measured with the objective of comparing it to the PC implementation and bounding the time the processing of an image was expected to take. These results were then compared to the ones obtained in Section 2.3.1.

In addition, execution time is related to the FAST threshold value. These parameters are indirectly related: Execution time of ORB depends on the input image and the ORB configuration, which, in this implementation, is limited to selecting a threshold value for the FAST algorithm. This threshold indirectly determines the number of feature points that are extracted, with less restrictive threshold values allowing for more feature points and vice versa. This impacts both execution time and memory usage of the iteration, as well as energy consumption.

Since the availability of RAM and FLASH memory on a microcontroller is low, memory usage was evaluated to identify potential constraints.

The procedure for measuring execution time and memory usage for each frame, processed one at a time, was performed as follows:

1. Each frame was converted to a byte array using a Python script on the PC.
2. The byte array was transmitted through UART from the PC to the microcontroller.
3. The microcontroller processed each frame using different FAST thresholds, starting from 255 and decrementing by 1. This process continued until either the threshold reached a value of 1 or a memory allocation failure ocurred due to an excessive amount of keypoints extracted from the frame.
4. Timestamps at the start and at the end of each function were recorded in millisecond resolution. These were transmitted through UART to the PC once the processing of the image finished.

**Figure 2:**
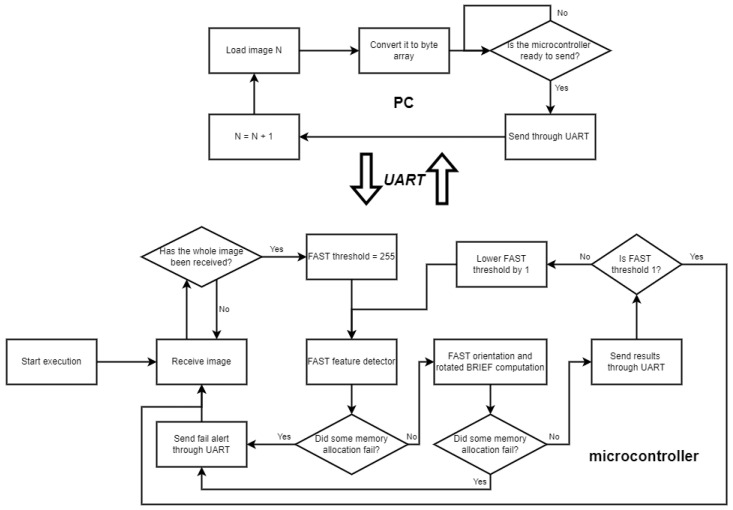
Flow diagram of the procedure for obtaining execution time measurements using the microcontroller.

This process was executed for each of the first 200 images of the dataset. A more detailed diagram of the process is shown in Figure 2.

Logs were analyzed to extract execution times, which were compared to the values obtained with the PC implementation. Execution times were also evaluated as a function of the FAST threshold and the number of features that the FAST function acquired.

Memory allocation failures were related to the FAST threshold that produced them. The reason for this is that, for a given image resolution, the factor that produces memory allocation failures is the number of feature points obtained by the FAST algorithm, which is indirectly set by the FAST threshold value. Whenever a memory allocation failure occurs for a certain threshold value, the next image is requested because less restrictive thresholds would also provoke a failure.

#### 2.3.3. Energy Consumption Measurements

The energy consumption of the microcontroller during image processing was measured with the objective of characterizing it in relation to different variables and the bound of the energy a process is expected to require. These measurements, along with execution time values, are essential to evaluate the viability of executing ORB on this specific microcontroller for specific low power constraints.

The energy measurement process is described as follows:

1. Each image is firstly converted to a byte array using an automated script.
2. The measurement unit is configured and started through the Standard Commands for Programmable Instruments (SCPI) interface using an automated script.
3. The byte array is transmitted through an Universal Asynchronous Receiver/Transmitter (UART) bus to the microcontroller.
4. The microcontroller processes the image using a fixed FAST threshold value of 50, recording timestamps for each function
5. Once the processing is finished, the microcontroller transmits the timestamps back to the PC through UART. Timestamps are measured in millisecond resolution.
6. Power consumption measurements are transmitted from the measurement unit to the PC via the Transmission Control Protocol (TCP) socket.

**Figure 3:**
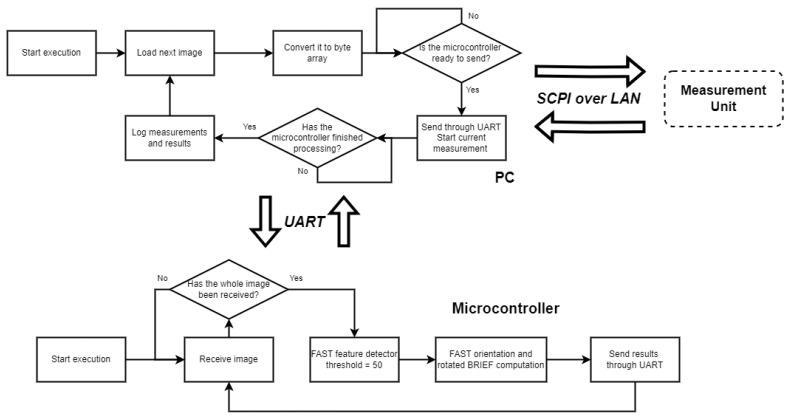
Flow diagram of the procedure for obtaining power consumption measurements using the microcontroller.

This process was executed twice for all images from the dataset. Each image was processed twice—once with the microcontroller powered at 1.8 and once at 3.3 volts for each image. The reason for these values is that the microcontroller supports supply voltages in the rage between 1.7 and 3.6 volts [19]. The voltage levels that were used are commonly used values. A more detailed diagram of the process is shown in Figure 3.

For each execution, current consumption was logged. Power consumption was calculated by integrating the product of the measured current and the supply voltage.

## 3. Results

### 3.1. Memory Usage Analysis

**Figure 4:**
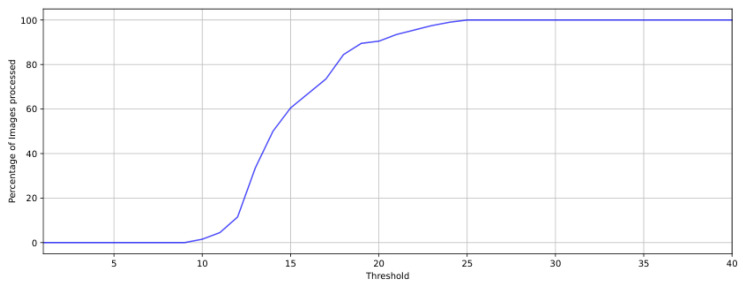
Cumulative distribution function of the images that are were to be processed by each FAST threshold. The y axis represents the percentage of images that could be processed for a given threshold without producing any memory allocation errors.

The cumulative distribution function (CDF) of the FAST thresholds for which no memory allocation failures occurred is shown in Figure 4.

Figure 4 shows that a threshold of 25 or higher allowed for the correct functioning of the program for all images. For thresholds below 25, memory allocation failures happened when processing some of the images, and below a threshold of 10, no image could be processed without errors. For the tests that followed, every combination of frame and threshold value that resulted in a memory allocation failure was discarded because the program stopped at the moment the failure occurred. In total, 200 images were considered for this test, which, combined with the presented distribution, allowed for 48,104 different combinations of image and threshold where memory constraints were satisfied. These combinations were then used in the following sections.

### 3.2. Execution Time Comparison Between PC and Microcontroller

**Figure 5:**
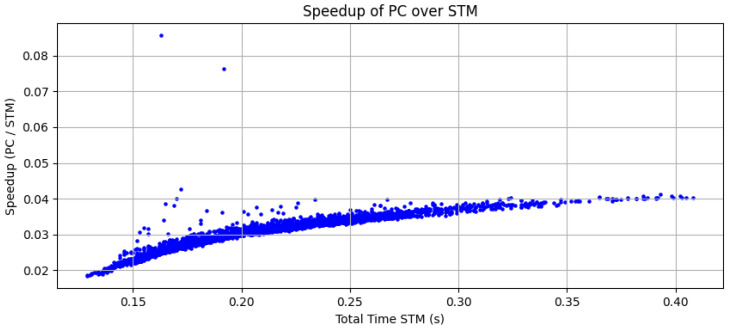
Speedup of the microcontroller with respect to the PC for ORB.

The execution times of ORB were compared between the microcontroller and the PC implementations. The results show a mean speedup of 0.03× on the microcontroller with respect to the PC. A plot of the speedup as a function of the execution time at the microcontroller (Figure 5) shows a tendency for lower speedup for lower execution times.

### 3.3. Number of Keypoints vs. Execution Time

**Figure 6:**
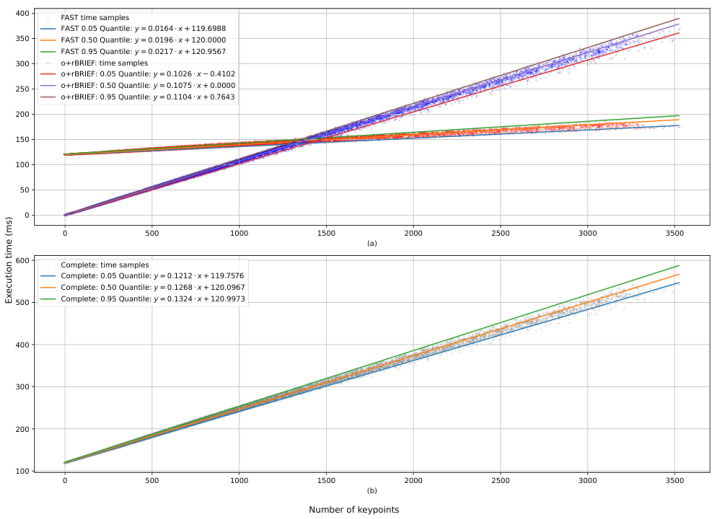
Execution times of each module with respect to the number of keypoints that FAST obtained. (**a**) Execution time for each module: FAST in red and o+rBRIEF in blue. (**b**) Total execution time: Each point represents an image and a specific threshold. The legend shows analytical functions for each regression.

This analysis characterizes how the number of points contributes to the run time of each part of the algorithm. For Figure 6a,b, the x axis represents the number of keypoints that one image has provided. The y axis represents the execution time of either each function in the case of Figure 6a; or the total time in the case of Figure 6b. In total, 48,104 samples were used, corresponding to the processing of the first 200 images of batch 0 with different values for the FAST threshold.

Figure 6a shows the execution time of the FAST and o+rBRIEF modules as a function of the number of keypoints detected by the FAST module, while Figure 6b shows the total execution time of the process in relation to the number of keypoints detected.

Two important aspects must be considered:

- The numerical regression of FAST depends on the image resolution, and it is expected to scale linearly to the number of pixels if the resolution varies.
- o+rBRIEF, however, depends only on the number of keypoints that were obtained, which means that the relationship between execution time of o+rBRIEF and the number of keypoints is the same for any image resolution and has the numerical values that are shown in Figure 6a.

For every plot, analytical functions for each quantile are shown with the purpose of bounding the expected execution times of each module.

### 3.4. Threshold vs. Execution Time

This analysis characterizes how the FAST threshold impacts the execution time of the process. In total, 48,104 data points have been considered for this analysis, accounting for 200 imagers per FAST threshold value. Figure 4 provides details on the distribution of images per threshold that was considered for this test. None of the sample points that were used in this analysis produced a memory allocation failure.

**Figure 7:**
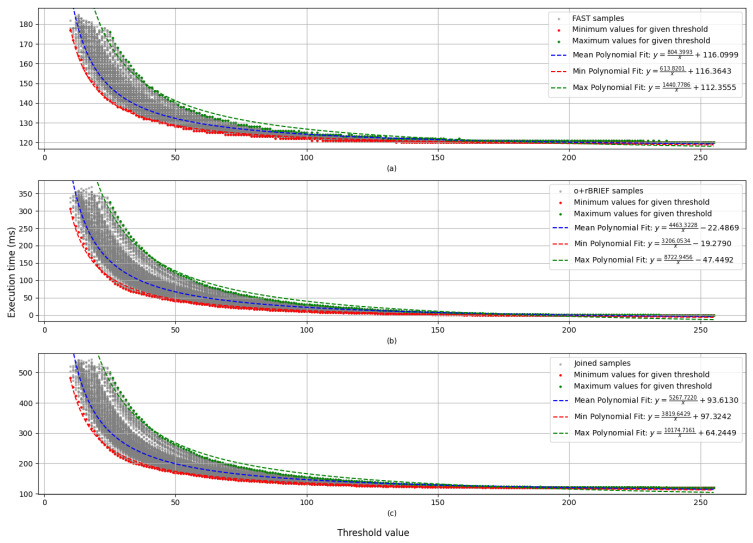
Execution times of each module with respect to the threshold value that was applied: (**a**) execution time for the FAST module; (**b**) execution time for the o+rBRIEF module; (**c**) total execution time. Each point represents an image and a specific threshold. The legend shows analytical function of each polynomial fit.

Figure 7 shows the relationship between execution time and the FAST threshold value.

Because the relation between the threshold and the execution time is not linear, interquantile regression was not applied. Instead, an inverse polynomial fit was applied to the mean, minimum, and maximum values of the execution times for each threshold value. For maximum times, threshold values below 25 were not taken into account due to the fact that not all images were able to be processed without memory allocation failures.

### 3.5. Energy Consumption

Energy consumption on the microcontroller was characterized in relation to execution time. All 4541 images from batch 0 of the KITTI dataset were processed using a fixed FAST threshold value of 50. Each image was processed twice, as stated in Section 2.3.3. In total, 9082 processes were performed for this test.

**Figure 8:**
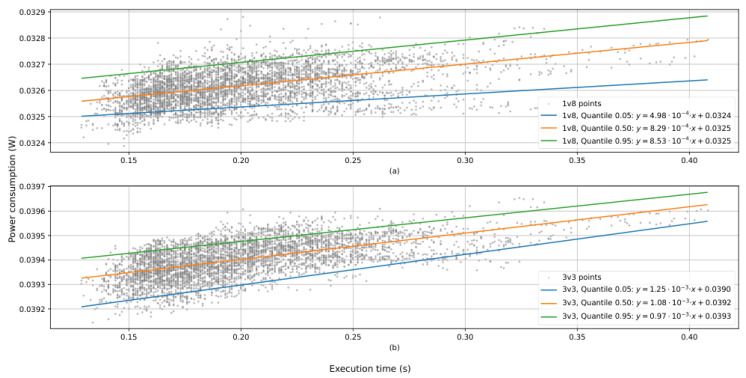
Power consumption of the whole process with respect to the execution time with supply voltage: (**a**) 1.8 volts; (**b**) 3.3 volts. Each point represents an image and a specific threshold. The legend shows analytical function for each regression.

Figure 8 shows the power consumption of the whole process, featuring FAST and o+rBRIEF combined.

Figure 8 reveals that the power consumption was not constant in relation to the execution time. The rate of change is both of the following:

- Low enough for a mean value to be representative.
- Noticeable enough to be further analyzed.

**Figure 9:**
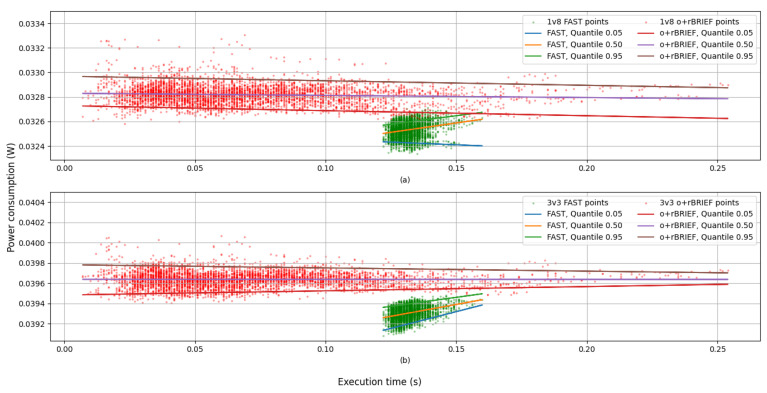
Power consumption of each module with respect to the execution time with supply voltage: (**a**) 1.8 volts; (**b**) 3.3 volts. Points in green correspond to FAST samples, and points in red correspond to o+rBRIEF samples.

Higher power consumption corresponding to greater execution times suggests that FAST and o+rBRIEF do not consume the same power. To confirm this hypothesis, Figure 9 differentiates the power consumption for each module.

Figure 9 shows that FAST consumed slightly less power in contrast to o+rBRIEF. Analytical expressions for quantiles are not provided due to the rate of change of power consumption with relation to execution time being of the order of microwatts per 100 ms. Instead, Table 1 presents mean and standard deviation statistics for the power consumption.

## 4. Discussion

The criteria for considering this solution viable for a SLAM solution is the existence of continuity between images, and this will relate directly to execution time. Power consumption is not considered a viability criteria because it is dependent of the specific use case as to whether the presented power consumption is suitable.

Figure 6 and Figure 7 show that the frame rate of the system is approximately 1.5 fps, even in the most restrictive settings. Since the restriction of execution time for visual odometry is continuity between frames, it is reasonable to consider this frame rate viable for some environments like human pace.

Figure 8 and Figure 9 show that the power consumption is of the order of tens of milliwatts. When compared to other works that offer hardware-accelerated solutions, only time experiences a significant decrease in performance.

The most important advantage of this solution is the economical one. The microcontroller that was used in this work (STM32U575ZIT6-Q) has a retail price of EUR 11.51. This is significantly more cost-effective than any ASIC or FPGA.

When compared to Table IV of [14], this work presents a performance per watt measurement of 154.3 for the whole batch 0 of the KITTI dataset (this measurement does not take into account the image resolution), with a FAST threshold value of 50. This is even better than every solution based on FPGAs, mainly due to the difference in power consumption.

It is also remarkable that it is not the purpose of this work to show the best capabilities of this microcontroller. No more optimizations than those explicitly specified were implemented. The microcontroller used includes built-in hardware modules that, if used, will improve execution speed. One example is the COordinate Rotation DIgital Computer (CORDIC) hardware accelerator to compute trigonometric functions, which are used in the o+rBRIEF module. Another example that could improve performance is the NeoChrom GPU that is available on other STM32U5 microcontrollers, with which the two modules described in this paper can be parallelized. The effects of implementing them are unknown, but it can be assumed that they would improve performance considerably.

Other microcontroller manufacturers may also offer other Cortex-M33-based microcontrollers that offer even more hardware improvements. Even other non-ARM-based microcontrollers may offer better execution times or lower power consumption.

These improvements and assumptions will further accentuate the point of this work: modern microcontrollers enable low-cost and low-power solutions in fields where hardware acceleration solutions have usually been the only option. The decrease in performance will make it unusable for several applications, especially those where the device movement is too fast for the presented frame rate for continuity between images to be maintained. However, in applications where movement is not particularly fast and cost is a factor to consider, a solution based on a low-power microcontroller may be adequate.

Use cases for this solution depend on one main criterion that affects the majority of visual odometry solutions: There must be continuity between consecutive frames. Due to how visual odometry works, feature points are related between frames in order to calculate the displacement between them. Therefore, this solution is suitable for use cases where feature points appear in at least consecutive frames, which in this case is around 1 s of separation in the worst case. These cases may include logistic systems, autonomous low-power vehicles, or wearable devices, as these systems require low power consumption and allow for the aforementioned timing constraints.

In case this solution is suitable, one relevant figure is how much a usual battery would last when executing the algorithm. For this matter, two different scenarios are considered to allow for approximations:

- Smartphone battery: A typical smartphone battery capacity is 4000 mAh at 3.7 volts, which accounts for 14.8 Wh. In this case, using the mean power consumption presented in Section 3.5 at 1.8 volts (32.6 mW) would result in a maximum execution time of approximately 454 h.
- Drone battery: Drones have a lower battery capacity compared to smartphones. A typical value is 2600 mAh at 3.7 volts, which accounts for 9.62 Wh. Using the same power consumption as before would result in a maximum execution time of 295 h.

These results, however, do not take into account other algorithms or peripherals that would be present in any location system, as well as efficiency in voltage conversion. These would even reduce the presented execution times drastically.

## 5. Conclusions

This work presented an alternative approach to ORB feature detection and description by leveraging modern microcontrollers. The performance is significantly lower in comparison to other more powerful platforms, with a 0.03× mean speedup compared to a PC, as shown in Section 3.2. However, the fact that a 1241 × 376 resolution image can be processed in less than 1 s demonstrates that this does not preclude their use in real-time applications.

Performance metrics, specifically execution time and power consumption, were evaluated using a publicly available image dataset. From the results, we may conclude that, given the execution times that were presented in Section 3.3, continuity between consecutive frames is feasible in some scenarios if the frame resolution is 1241 × 376 or lower. Furthermore, the power consumption was measured to be 32.6 milliwatts when the microcontroller was powered at 1.8 volts. This resulted in being more energy-efficient than some FPGA-based solutions, as discussed in Section 4, thus making this solution well suited for energy-constrained applications.

Future work may explore the integration of stereo image processing to enable scale estimation or using hardware accelerators embedded in the microcontroller, further expanding the capabilities of this approach.

## Figures and Tables

**Table 1:** Power consumption statistics considering functions and the whole process independent of the execution time.

Function	Mean Power Consumption @ 1.8 V (W)	Standard Deviation @ 1.8 V	Mean Power Consumption @ 3.3 V (W)	Standard Deviation @ 3.3 V
FAST	0.032527	0.000064	0.039302	0.000067
o+rBRIEF	0.032819	0.000081	0.039638	0.000079
Whole program (FAST and o+rBRIEF)	0.032616	0.000071	0.039405	0.000078

## Data Availability

Data are contained withing the article.

## Abbreviations

The following abbreviations are used in this manuscript:

SLAM	Simultaneous Location And Mapping
FAST	Features from Accelerated Segment Test
BRIEF	Binary Robust Independent Elementary Features
ORB	Oriented FAST and Rotated BRIEF
FPGA	Field Programmable Gate Array
ASIC	Application-Specific Integrated Circuit
LiDAR	Light Detection and Ranging
PC	Personal Computer
SRAM	Static Random-Access Memory
SCPI	Standard Commands for Programmable Instruments
TCP	Transmission Control Protocol
CORDIC	COordinate Rotation DIgital Computer
